# 

**Published:** 1850-05

**Authors:** 


WHOLE SERIES—VOL. VII, NO. I.

VOL. III.] MAY, 1850. [No. 1.

### THE NORTH-WESTERN

### MEDICAL AND SURGICAL

### JOURNAL.

EDITED BY

JOHN EVANS, M.D.,

PROFESSOR OF OBSTETRICS ETC., IN RUSH MEDICAL COLLEGE.

AND

EDWIN G. MEEK, A.M., M.D.

PUBLISHED EVERY OTHER MONTH.

AT TWO DOLLARS PER ANNUM IN ADVANCE.

CHICAGO AND INDIANAPOLIS:

C. A. SWAN, PRINTER.

1850.

ENLARGED SERIES—VOL. V, NO. I.

